# A model for point of care testing for non-communicable disease diagnosis in resource-limited countries

**DOI:** 10.1017/gheg.2019.6

**Published:** 2019-09-11

**Authors:** Stuart Malcolm, Joane Cadet, Lindsay Crompton, Vincent DeGennaro

**Affiliations:** 1 Department of Internal Medicine, Emory University School of Medicine, 80 Jessie Hill Jr. Dr. SE, Atlanta, GA 30303, USA; 2 Department of Medicine, University of Florida College of Medicine, Gainesville, USA; 3 London School of Hygiene and Tropical Medicine, London, United Kingdom of Great Britain and Northern Ireland; 4 Innovating Health International, Port-au-Prince, Haiti; 5 Florida International University College of Medicine, Miami, FL, USA

**Keywords:** Chronic disease, global health, Haiti, non-communicable disease, point of care

## Abstract

Non-communicable disease diagnosis frequently relies on biochemical measurements but laboratory infrastructure in low-income settings is often insufficient and distances to clinics may be vast. We present a model for point of care (POC) epidemiology as used in our study of chronic disease in the Haiti Health Study, in rural and urban Haiti. Point of care testing (POCT) of creatinine, cholesterol, and hemoglobin A1c as well as physical measurements of weight, height, and waist circumference allowed for diagnosis of diabetes, chronic kidney disease, dyslipidemias, and obesity. Methods and troubleshooting techniques for the data collection of this study are presented. We discuss our method of community-health worker (CHW) training, community engagement, study design, and field data collection. We also discuss the machines used and our quality control across CHWs and across geographical regions. Pitfalls tended to include equipment malfunction, transportation issues, and cultural differences. May this paper provide information for those attempting to perform similar diagnostic and screening studies using POCT in resource poor settings.

## Introduction

Non-communicable disease (NCD) are the leading cause of death worldwide [1] and cardiovascular disease is the leading cause of death and disability-adjusted life years lost in low-and middle-income countries. In the Caribbean, chronic diseases are now the leading cause of premature mortality, accounting for nearly half of all deaths of persons under 70 years, and for two out of three deaths overall [2]. Therefore, proper measurement of these biochemical measurements in defining disease prevalence is necessary. Point of care testing (POCT) offers an avenue for screening in resource-limited settings.

NCD diagnosis frequently relies on biochemical measurements. The infrastructure required for biochemical testing is unavailable in many regions, especially in low-income countries. In high-resource settings, blood tests are sent to laboratories where cooling and specific storage requirements are in place; they also have reporting systems to central databases that often require internet connection, as well as trained individuals to draw blood, monitor quality, and report results. Testing storage during transport may be economically too difficult if distances are far and hard to traverse. Laboratories themselves may lack electricity, refrigeration, certain equipment, and biomedical technicians for repairs amongst other barriers. Additional issues for patients include poor road infrastructure, financial resources, distance from doctors or laboratories, and lack of understanding of disease processes.

Haiti, like many low-income countries, has low literacy levels [3] and low health literacy [4]. Most patients are not diagnosed at all, or at advanced stages of chronic illnesses when symptoms are prevalent, or are not diagnosed according to guidelines as there is little primary care for screening. In low-income countries with severe resource constraints, innovative methods for efficient screening and diagnosis are essential for the nearly one billion people worldwide who will require daily medications for NCDs and their risk factor management by 2030 [5].

POCT has been well validated as comparable to testing in laboratory settings for hemoglobin A1c (HbA1c) [6, 7], creatinine [8], and cholesterol (total, high-density lipoprotein cholesterol (HDL-C), triglycerides (TGs)) [9, 10]. POCT could be potentially used for screening of illnesses (for example, how dyslipidemia or diabetes is screened for in the United States). One can imagine that not having to go to a local laboratory away from a clinic or having instant results to a patient may increase desire for testing. POCT has been shown to increase the testing of specific diseases when available, for example when rapid HIV tests were made accessible [11]. An example of monitoring control on treatment would be diabetes. Using HbA1c to monitor a disease state every three months reduces the need for self-testing of daily blood sugars and can be especially helpful where resources don't permit a daily measurement.

The use of community-health workers (CHWs) for management of NCDs has been shown to be effective for prevention [12], diagnosis [13], and management [14, 15] of NCDs [16], including multi-component interventions [17], and in low-resource settings [18–20]. Using CHWs is ideal in many resource-poor settings which lack physicians and other more highly skilled healthcare providers. For example, women's cancer awareness research conducted in Haiti by Innovating Health International discovered that CHWs are one of the top two most-trusted sources of health information in the country, above even doctors [21].

We present a model for using POCT to determine disease prevalence as used in our study of chronic disease in the Haiti Health Study [22], in rural and urban Haiti. We hope that this paper provides useful guidance for those attempting to perform prevalence or screening studies using POCT in resource poor settings.

## Materials and methods

### Community engagement

Knowing that local buy-in was of tantamount importance, prior to initiation of the study existing CHWs announced to local community leaders (priests, government officials, school principals, etc.) the study aims and sought their input and acceptance. These community leaders then relayed this information to their respective groups and answered any questions about the study. This was relayed back to the study team and through a back and forth process, concerns were addressed to help minimize issues and increase buy-in from the community.

### CHW enrolment and training

The CHWs employed for the study were established members of their communities, but were taken from outside of the study site so as to limit concern for knowing study participants. In addition to general CHW training prior to the study, the CHWs received data collection training over a period of 2 weeks. The initial training sessions were dedicated to understanding the goals of the study and general research principles such as privacy, confidentiality, safety, and the importance of obtaining consent. The format of these sessions was primarily group discussions. Familiarity with the modified World Health Organization (WHO) Stepwise Approach to Surveillance (STEPS) survey was established through role play, with the CHWs acting as both participants and data collectors. The trainers also discussed interview techniques and examples of how the CHWs could overcome obstacles encountered when collecting data. Trainers also observed pairs of CHWs as they completed the survey and provided direct feedback as needed. The POCT materials were introduced with a basic overview of their purpose and the diseases they are associated with. The execution of the POCT was taught using a teach-back technique. Prior to the completion of the training, all CHWs completed practice interviews with members in the community while being observed by the trainers. The selection of the CHWs to ultimately complete the study was based on the demonstration of proficiency in conducting the survey and implementing all of the POCT.

### Study design

Using a multi-stage cluster sampling method, six CHWs recruited more than 2100 participants door-to-door and collected data on diet, exercise, education, cell phone usage, income, NCD risk factors, and mental health using a modified WHO Stepwise Approach to Surveillance (STEPS) survey [23]. Measurements of height, weight, waist circumference, blood pressure, and POCT for creatinine, cholesterol, and HbA1c were also collected. POC fingerstick tests for HbA1c, lipids, and creatinine were conducted by CHWs during the same visit in the Haiti Health Study [22].

Men and women between 25 and 65 years of age, residing in Rivière Froide, Carrefour and Cabral of Thomonde, Central Plateau were eligible to participate in the study. Men and women who were cognitively impaired or women who were pregnant were excluded. The Institutional Review Board approval was obtained through the University of Florida College of Medicine and the Haitian Medical Association National Bioethics Committee.

### POCT data collection

CHWs introduced the project to participants, which was generally well-received. Each of the POCT fingerstick tests was obtained in a similar manner, starting with proper preparation and cleaning of the site of sampling with an alcohol swab. Two to three fingersticks were required for the three tests. It was too logistically difficult to only visit households that had fasted that day, especially since measurements were conducted well into the afternoon, thus tests were considered random. Initially, each biochemical test was performed with a single fingerstick to a separate finger, but this was discovered to be cumbersome to the participants. As a result, the creatinine test was performed on a second drop of blood from the same fingerstick after either the HbA1c or cholesterol test was completed without affecting the results, as per the manufacturer's specifications. The decreased burden on the participants led to an increase in acceptability as per the CHWs, although not statistically significant. The exposed blood was then drawn from the tip of the finger, making sure to avoid massaging the finger per guidelines from the devices and fed into respective machines.

### Devices

Creatinine, as measured via StatSensor Creatinine Express device (Nova Biomedical, Waltham, MA), was conducted via a drop of blood fed into an electronic device which would record the value. HbA1c was obtained using A1c Now+ (Polymer Technology Systems, Inc., Indiana, US) which required mixing of a blood sample with a small reagent contained within the device packet and then eventual deposition of the material onto a membrane reading chip. The lipid testing was conducted with the CardioChek Lipid Test (Polymer Technology Systems, Inc., Indiana, US) which required blood deposition on a membrane that was read using a laser detection system; total cholesterol, TGs, and HDL-C were recorded. All tests were carried out via manufacturer guidelines. Results obtained from the electronic testing device were recorded on paper charts and then transferred to electronic database RedCAP [24]. All sharps utilized during testing were placed in portable sharp-containers, some as simple as hard plastic bottles, and disposed of at local health clinics that had the proper capacity to manage these materials. Biohazardous materials such as used gloves and gauze were disposed in plastic bags that CHWs carried with them during the day and emptied each evening.

All testing equipment, which included biochemical tests as well as a weight scale, tape measurer, blood pressure cuff, disposal equipment, and icepacks were contained in portable backpacks that the CHWs carried with them during the entirety of the study. Using this equipment and model, vast distances of land could be traversed despite the packs weighing slightly over 20 pounds.

### Quality control

Quality control was conducted throughout the testing period. Periodic checks of entered data into the paper forms were compared with recorded values in the cholesterol and creatinine measuring devices to assure fidelity between paper forms and recorded values. Lipid assays were compared to set a standard assay. HbA1c was compared to healthy subjects with known values as validation.

### Disease definitions

Diabetes was diagnosed using HbA1c ⩾ 6.5% and prediabetes as defined by HbA1c ⩾ 5.7% to <6.4%, as per the American Diabetes Association. Chronic kidney disease (CKD) was defined using point of care (POC) creatinine which when combined with patient's weight, height, age, and sex is used to calculate glomerular filtration rates (GFR) (using the Cockroft–Gault calculation) for each patient. The GFR for stage 1 CKD is ⩾90 ml/min, stage 2 is 60–89 ml/min, stage 3 is 30–59 ml/min, stage 4 is 15–29 ml/min, and stage 5 is <15 ml/min. POCT of cholesterol, which included separate results for total cholesterol, HDL-C and TGs, and calculated low-density lipoprotein cholesterol (LDL-C), were used to define dyslipidemias. Elevated total cholesterol was defined as ⩾240 mg/dL, TG as ⩾150 mg/dL, low HDL-C as ⩽50 mg/dL in women and ⩽40 mg/dL in men, and elevated LDL-C as ⩾130 mg/dL. Metabolic syndrome was defined using modified-Adult Treatment Panel III (ATPIII) criteria which we defined by three of the following: HbA1c > 5.0%, waist circumference ⩾102 cm (men) or ⩾88 cm (women), blood pressure ⩾130/85 mmHg, TG level ⩾150 mg/dl, HDL-C cholesterol level less than 40 mg/dl (men) or 50 mg/dl (women). Traditional ATPIII criteria do not use HbA1c but rather the diagnosis of diabetes or fasting blood glucose (FBG) ⩾100 mg/dL. We were limited in being unable to use FBG in our definition due to logistical constraints. We chose HbA1c of 5.0% to correlate roughly with FBG of 100 mg/dL. From prior studies, HbA1c of 5.0% correlates with a FBG between 96.8 mg/dL [25] and 118.7 mg/dL [26].

Standardized automated wrist blood pressure monitors (Omron Series 7) were included in our study to measure blood pressure. Wrist blood pressure measurements were used because of variability of techniques between users and burden of bringing multiple-sized blood pressure cuffs with the already large volume of materials for the study.

## Results

Over 2100 participants partook in the study. Between 14.1% and 17.1% were hypertensive, 16.4% to 23.1% were diabetic, and 10.5% to 14.2% had CKD, depending on the region. Metabolic syndrome was found in 26.3% of the population. Detailed patient demographics and results by sex and location are presented below and are adapted from DeGennaro *et al*. [22].

Of all those tested (*N* = 2104), 6.1% (*N* = 129) had a new, previously unknown diagnosis of hypertension, which means that 39.3% of all hypertensives (*N* = 328) did not know of their diagnosis previously. Of those who screened positive for diabetes (*N* = 366), virtually all (93%) were unaware of their diagnosis (*N* = 342). Of the total population tested, 2.39% (*N* = 43) had high-total cholesterol, and 34.7% (*N* = 631) had low HDL-C, all uncontrolled, since only five patients had known high-total cholesterol (Tables 1 and 2).

**Table 1.** Socio-demographic characteristics of study participants

	Rural (*n* = 706)		Urban (*n* = 1425)		Total (*n* = 2131)	
Variable	42.4	(s.d. 11.7)	39.9	(s.d. 11.7)	40.8	(s.d. 11.8)
Mean age (s.d.) (years)	*n*	%	*n*	%	*n*	%
Sex						
Male	258	36.5	571	40.1	829	38.9
Female	448	63.4	854	59.9	1303	61.1
Monthly income (US$)						
<25	211	30.1	206	14.7	417	19.8
25–50	89	12.7	144	10.2	233	11.1
51–250	194	27.7	357	25.5	551	26.2
251–500	177	25.2	544	38.8	721	34.5
>500	29	4.1	148	10.5	177	8.4
Education						
No formal schooling	559	85.3	715	56.9	1274	66.6
Less than primary school	67	10.2	332	26.4	399	20.8
Primary school	19	2.9	173	13.7	192	10.5
Secondary school	5	0.7	25	1.9	30	1.5
University	5	0.7	11	0.8	16	0.8

**Table 2.** Results from the Haiti Health Study

	Prevalence by sex			Prevalence by location		
	Males	Females	*p* values^a^ (sex)	Urban	Rural	*p* values^b^ (location)
Hypertension (*n* = 2104)	11.0	20.2	<0.001	17.1	14.1	0.185
Diabetes (*n* = 1858)	18.6	20.8	0.001	16.4	23.1	<0.001
CKD^c^ (*n* = 1980)	8.8	15.8	<0.001	14.2	10.5	0.086
High total cholesterol	0.4	4.0	0.000	2.7	1.8	0.037
High LDL-C (*n* = 1819)	0.8	4.5	<0.001	3.3	2.1	0.203
Low HDL-C (*n* = 1819)	35.8	33.6	0.100	32.3	36.2	0.094
High TGs (*n* = 1819)	5.9	9.0	0.001	7.1	7.8	0.521
Overweight and obese ^d^ (*n* = 2074)	14.3	34.3	<0.001	30.4	18.2	<0.001
Large waist circumference (*n* = 2074)	4.2	34.3	<0.001	30.4	18.2	<0.001

a Comparing male and female prevalence.

b Comparing urban and rural prevalence.

c CKD stages 3–5 according to the Cockcroft–Gault formula for the GFR.

d BMI defined as ⩾25 kg/m^2^.

We purchased and used 2500 test strips to perform testing on the population of just over 2100 individuals. The wastage included training, error codes, and failure to record the results. The POCT average costs for this study will be different than what a program might need to use in a practical setting since the home visits took over an hour, much more time than a CHW would need for disease diagnosis or monitoring when used clinically. However, the equipment and consumables for POCT can be calculated and extrapolated. Hypertension, body-mass index (BMI), and waist circumference testing costs were negligible because equipment was reused. Cholesterol cost was $8.57(USD) per test, HbA1c cost was $6.52 per test, and creatinine cost was $3.40 per test. When all the other costs, such as gloves, gauze, and disposal were included, the total equipment and consumable costs per participant was $19.03. The HbA1c machines come bundled with 20 test strips, but the creatinine and cholesterol machines cost $200 and $500 each, respectively. The total cost per participant, or patient, will drop the more the machines are used over time and the more the training costs diminish in significance for populations larger than 2100 people.

## Discussion

Our study revealed a high burden of chronic disease in both urban and rural Haiti. Hypertension was relatively well diagnosed, which may be attributed to the fact that this is diagnosed with blood pressure cuffs rather than blood tests. Untreated diabetes and dyslipidemia can lead to worse morbidity and mortality and these diseases can be treated successfully with oral medications. POCT can both be used as an epidemiologic tool but also a screening test for clinics to ensure proper diagnosis for patients and thus reduce this burden.

### POCT issues

POCT results are limited by their design and their testing environment. Although well-verified in the literature, there have been discrepancies noted between tests. Of note, kidney function assays have been shown to have higher discrepancies at creatinine <1.4 mg/dL, although they are generally regarded as accurate [27]. Creatinine measurement in whole blood is challenging as it can be influenced from different disease states (e.g. hyperglycemia, uremia, and treatment medications) that may be difficult to measure in resource-poor settings. The CardioChek device used in our study has been well-verified [28].

POC tests are most accurate using the standards for which they were designed. Most POC tests are designed for primary care facilities in high-income nations which are often temperature regulated. For example, each POCT device required certain temperature ranges and are programmed to read error codes when ambient temperatures are out of range. During summer data collection in Haiti, temperatures consistently exceeded 35°C which necessitated carrying the POCT device in bags packed with ice. This was initially quite cumbersome and required trial and error in the amount of ice to pack and occasionally they were too cold. To negate this, we used cloth packing to separate the machines from direct contact with ice. As the day progressed, the CHWs would empty out the water from melted ice to ensure a temperature range that was acceptable. Over time, the CHWs were able to devise a method such to maintain the tests during the day so that they would all work properly and consistently. To assure accurate POCT results, we periodically tested each POCT against subjects with known laboratory confirmed results. We felt that using a standard while in the field required excess space and cooling. Alternatively, testing while at our study storage facility would have been possible but we felt that this didn't reflect the temperatures inflicted upon the testing devices while out in the field.

Potential pitfalls include variation in tested variables. Known problems associated with testing for HbA1c include hemoglobin variants that increase variability in results. In our study, even the high-end prevalence estimates of hemoglobin variants (13.97%) did not affect the diabetes prevalence in aggregate [29]. However, a difference of one percent in HbA1c in an individual patient with a hemoglobin variant can be significant in a clinical setting, such as 6.4 mg/dl to 7.4 mg/dl is the difference between needing treatment for diabetes and not. With creatinine, there is always a possibility that patients may be dehydrated at the time of testing, and perhaps this may be more likely for manual laborers in the field as opposed to when they visit a clinic for standard laboratory testing. POCT might increase the chances that patients have eaten more recently; however, recent guidelines suggest that eating may not have a great impact on lipid panels [30]. Having proper knowledge of the limitations of testing equipment is important in any epidemiological study.

### Survey conditions

Other logistical problems that arose from door-to-door POCT were the vast distances required for healthcare professionals to get to rural communities as well as the carrying of equipment that houses and protects the tests. This required CHWs with a strong baseline level of physical fitness, especially in the mountainous regions. Although the backpacks had all the appropriate amount of testing equipment and ice packs, the backpacks were substantially heavy and work was difficult. Paper surveys for the study contributed to much of the weight. Additionally, since we measured BMI, scales were carried to each household and added significantly to the weight and size of the load that each CHW had to carry. Through trial and error CHWs were eventually able to estimate the amount of materials needed through the day and made data collection easier. The use of motorcycles was less expensive means to transport CHWs and testing materials up narrower roads that alleviated some of the fatigue associated with carrying equipment. The backpacks could be easily worn while sitting on the back of a motorcycle taxi, ideal for low-income and remote settings. When motorcycles could go no further, much of the data collection was done on foot.

One experience noted from our study in Haiti was the cultural fear of losing too much blood via blood testing, which would result in ‘weakness’. POCT was thus ideal as it minimized the amount of the sample needed for adequate testing. Most studies have indicated that POCT is favored, or at least regarded without difference to, traditional venipuncture [29]. It also had the added benefit of decreased need for training in this procedure, transport, and storage of blood vials.

### POCT costs

There is a dearth of POCT used for epidemiological surveys and screening of NCDs. Our study offers a model for being able to measure chronic disease in low-income countries. POCT is thought to be more expensive that central laboratory testing [29], but central testing often relies on labor costs of nursing and ancillary staff which may be more expensive and which may not apply to rural settings that require follow-up after a certain amount of time. For example, for HbA1c and cholesterol for the NHS in the UK, it is more cost effective to utilize POC rather than traditional laboratory methods when considering missed appointments and other factors in the primary care setting [29]. In establishing effective strategies in low-infrastructure settings, the initial cost of POCT may be worth the initial investment. However, more specific analysis for particular settings (cost of transport, rate of missed clinic appointments, likelihood of follow-up, etc.) for each community would be required to establish the exact difference in cost. Considering infrastructure start-up costs in developing nations, costs may be significantly less, allowing for easier treatment initiation. Given the higher per test cost, scale-up to national treatment goals may not depend on POCT alone, but rural areas are the obvious beneficiaries of such a strategy.

Because NCDs require accurate lab data to effectively monitor treatment, the ability to remotely measure these values would be of incredible use. One could imagine that visiting a healthcare professional both entailed assessment and discussion of up-to-date lab tests would be most useful and perhaps engage patients to improve treatment adherence compared to standard laboratory testing that may require significantly more waiting time. For example, monitoring of HbA1c in an urban clinic in South Africa has shown that through POC HbA1c testing, the number of diabetes patients achieving optimal glycemic control increased by 125% and the number with very poor glycemic control halved [31]. In a study of remote Australian Indigenous communities, POC HbA1c leads to improve the time to follow-up with the physician and increase the frequency of HbA1c measurements per person over the year when compared to laboratory-based HbA1c testing. The use of POC HbA1c testing also showed improved glycemic control over a period of 15 months in this community [32].

### Advantages and limitations of model

Using CHWs for screening and diagnosis of NCDs has several advantages. In addition to the wide acceptance of POCT and CHWs used in the house, instant results of POCT offer the chance for immediate education by CHWs. All participants in our study received their results on a ‘Know Your Numbers’ card which allowed them to review results, understand normal and abnormal values, ask questions of the CHWs, and bring results to their doctor. For future programs, CHWs using POCT offer the possibility of immediate medication refills for those with well-controlled disease. CHWs could also call doctors at a clinic to discuss results and even possibly change medications in the community as well, although this is currently outside the scope of practice of CHWs in Haiti. CHWs are the trusted source of information in Haiti and many developing countries and the human interaction surrounding POCT may increase service utilization [33] and possibly treatment adherence [34], but this requires further study.

A limitation of the study model is based on the under-representation of men among our sample: 39.5% of the data came from men, and 60.5% from women. Since the study was conducted during the day and at homes, men may have been at work and thus less likely to have completed the study if they were selected. This has been cited as a limitation in other surveillance work in Haiti [35]. It is unknown if men are more averse to POCT. Since the study was cross-sectional, we did not follow up with participants to see if they sought and were started treatment once made aware of their diagnosis. Strengths of this study include the vast distance of an area that is extremely limited by infrastructure, which we felt was an important nuance that POCT provides. This is one of the few studies looking at chronic disease in Haiti. We hope that the design and troubleshooting experienced in our study may help in further establishment of other projects that will allow proper definition of prevalence and potentially screening of chronic diseases worldwide.
